# Enantioselective Quantification of Amphetamine and Metabolites in Serum Samples: Forensic Evaluation and Estimation of Consumption Time

**DOI:** 10.3390/metabo11080521

**Published:** 2021-08-06

**Authors:** Moritz Losacker, Michael Kraemer, Alexandra Philipsen, Kristina Duecker, Nadine Dreimueller, Jan Engelmann, Joerg Roehrich, Cornelius Hess

**Affiliations:** 1 Department of Forensic Toxicology, Institute of Legal Medicine, University Medical Center Mainz, Am Pulverturm 3, D-55131 Mainz, Germany; roehrich@uni-mainz.de (J.R.); c.hess@spmd-rfb.de (C.H.); 2 Department of Forensic Toxicology, Institute of Legal Medicine, University Hospital Bonn, Stiftsplatz 12, D-53111 Bonn, Germany; michael.kraemer@uni-bonn.de; 3 Clinic and Polyclinic for Psychiatry and Psychotherapy, University Hospital Bonn, Venusberg Campus 1, D-53127 Bonn, Germany; alexandra.philipsen@ukbonn.de (A.P.); kristina.duecker@ukbonn.de (K.D.); 4 Department of Psychiatry and Psychotherapy, University Medical Center Mainz, Untere Zahlbacher Str. 8, D-55131 Mainz, Germany; nadine.dreimueller@unimedizin-mainz.de (N.D.); jan.engelmann@unimedizin-mainz.de (J.E.); 5 Reference Institute for Bioanalytics, Friesdorfer Str. 153, D-53175 Bonn, Germany

**Keywords:** amphetamine, enantiomers, stereoselective, blood serum, chiral chromatography, liquid chromatography, mass spectrometry, forensic science, consumption time, driving under the influence of drugs (DUID)

## Abstract

In forensic toxicology, amphetamine intoxications represent one of the most common case groups and present difficult questions for toxicologists. Estimating the time of consumption and the current influence of the stimulant is particularly difficult when only total amphetamine concentrations are considered. Stereoselective analysis and the consideration of metabolites can provide valuable information to facilitate interpretation. An enantioselective liquid chromatography−tandem mass spectrometry (LC-MS/MS) method for detection of amphetamine, norephedrine and 4-hydroxyamphetamine was developed. Validation showed satisfactory selectivity, sensitivity, linearity (0.5–250 ng/mL), precision and accuracy for all enantiomers. The method was applied to a collective of 425 forensic serum samples and 30 serum samples from psychiatric inpatients stating their last time of amphetamine consumption. Norephedrine and 4-hydroxyamphetamine were detected more frequently at higher amphetamine concentrations and at lower amphetamine (R)/(S) concentration ratios, possibly indicating recent consumption. Mean (R)/(S) ratio of amphetamine was 1.14, whereas higher ratios (mean 1.36) were found for amphetamine concentrations below 100 ng/mL. The (R)/(S) ratios of psychiatric inpatients significantly correlated with the reported time intervals to last consumption. The use of amphetamine (R)/(S) ratios and the simultaneous detection of metabolites are promising factors that can facilitate estimation of consumption time and current impairment.

## 1. Introduction

Amphetamine remains one of the most frequently abused drugs worldwide [1]. Intoxications with this stimulant represent one of the most common case groups and present difficult questions for toxicologists [2,3,4].

Amphetamine is a sympathomimetic amine that increases brain levels of norepinephrine, serotonin and dopamine by promoting release and inhibiting reuptake of these neurotransmitters [5,6]. Typical acute effects after amphetamine use are euphoria, enhanced attention and alertness, increased psychomotor performance and loss of appetite. At higher doses, adverse effects such as agitation or lack of concentration can occur [3,7]. Excessive amphetamine use can result in serious intoxication and death, predominantly by acute cardiac or cardiopulmonary failure, cerebrovascular hemorrhage or hyperthermia [8,9,10]. The acute phase lasts about 6 h [11] and is followed by the subacute phase, which is characterized, e.g., by fatigue, loss of attention and depressive mood [3,12,13]. Due to the broad spectrum of effects, a forensic assessment of current impairment is particularly relevant in cases of driving under the influence of drugs (DUID) [3,10,14]. The determination of the absolute serum amphetamine concentration hardly allows a statement on the time of consumption and thus on the current influence of the stimulant [15]. This is due to differences in quantity and frequency of dosing due to habituation effects [16], as well as interindividual differences in metabolism [17,18].

Amphetamine is a chiral molecule and exists in the enantiomeric forms (S)-amphetamine (d-(+)-amphetamine, dexamphetamine) and (R)-amphetamine (l-(-)-amphetamine, levoamphetamine). Analytical chiral separation is most commonly conducted via capillary electrophoresis (CE) [19,20], direct [4,21] and indirect high-performance liquid chromatography (HPLC) [22,23] and indirect gas chromatography (GC) [24,25].

Studies suggest an approximately 3–4-fold higher efficacy of the (S)-enantiomer [26,27]. Studies further demonstrate stereoselective metabolism of amphetamine, with preferential elimination of the (S)-enantiomer [28,29,30]. In the human body, amphetamine is metabolized to norephedrine and 4-hydroxyamphetamine. Pharmacokinetics dependent on the absolute configuration are also reported for these metabolites, although hardly any data are available for the human blood [30,31,32,33]. It has already been suggested that the time course of (R)/(S) concentration ratios of stimulants can be used to draw conclusions on the time of consumption [34,35,36]. More sophisticated studies, especially on larger sample sizes, are scarcely available in the literature, and the potential of stereoselective analytics for forensic toxicology has not yet been exploited.

In this study, a stereoselective LC-MS/MS method for the detection of the enantiomers of amphetamine, norephedrine and 4-hydroxyamphetamine was developed and validated. The method was applied to the analysis of 425 forensic serum samples and 30 serum samples from psychiatric inpatients who were hospitalized voluntarily and reported their last time of consumption. The aim of this study was the forensic evaluation of the determined plasma concentrations with particular focus on the estimation of the time of consumption.

## 2. Results

### 2.1. Method Validation

**Figure 1:**
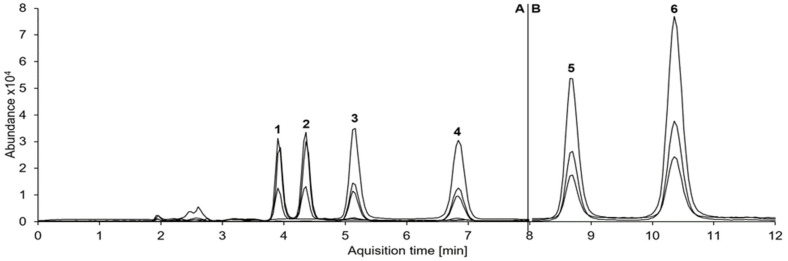
Multiple reaction monitoring (MRM) chromatograms for (R)-4-hydroxyamphetamine (1), (S)-4-hydroxyamphetamine (2), (1S,2R)-norephedrine (3), (1R,2S)-norephedrine (4), (R)-amphetamine (5) and (S)-amphetamine (6) at concentration levels of 20 ng/mL per enantiomer. MRM was split into two segments (A, B) at 8.0 min to reach a higher abundance. The total run time was 19 min. The following ion transitions are displayed in decreasing order of intensity (target ion underlined): 4-hydroxyamphetamine 152.1 → 107.0, 135.0, 77.0; norephedrine 152.1 → 134.0, 117.0, 115.0; amphetamine 136.1 → 91.0, 119.0, 65.0.

**Figure 2:**
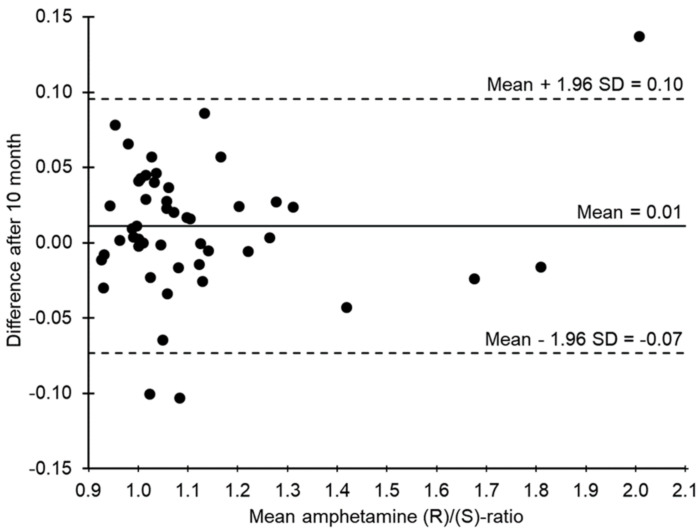
Bland-Altman plot of the stability of amphetamine (R)/(S) ratios after reanalysis after 10 months (n = 50). The *x*-axis shows the mean value of the two determinations and the *y*-axis shows the absolute deviation after 10 months.

Baseline separation was achieved for all analyte and internal standard enantiomers (Figure 1). Blank and zero samples showed no interfering signals. Cathine, on the other hand, coeluted with (1S,2R)-norephedrine. A linear calibration model without weighting could be used for quantification of all analytes (calibration range 0.5–250 ng/mL). For all analytes, precision (relative standard deviation (RSD)) and bias data at the lowest calibration level (0.5 ng/mL) were within the acceptable limits of the guideline, so this was accepted as the lower limit of quantification (LLOQ). Results for the external serum round-robin test indicated racemic amphetamine and were in good accordance with the manufacturers’ requirements. For enantiomers of 4-hydroxyamphetamine and norephedrine, the 0.5 ng/mL level was also the limit of detection (LOD); for amphetamine enantiomers, 0.1 ng/mL was accepted as LOD, although only quantifiable results (≥0.5 ng/mL) were considered for this study. For all analytes, results of further validation experiments were within acceptable limits (Table 1). For enantiomers of amphetamine and norephedrine, slight ion suppression at the low concentration level and slight ion enhancement at the high concentration level were observed. For 4-hydroxyamphetamine enantiomers, on the other hand, results for ion suppression (up to 78.7 ± 10.3%) and ion enhancement (up to 114.8 ± 6.7%) were more diverse. Recovery was rather low for enantiomers of amphetamine (mean 29.8%) and even lower for norephedrine (mean 12.0%) and 4-hydroxyamphetamine (mean 12.4%). For amphetamine-d11 and norephedrine-d3, results for recovery (maximum deviation 3.7%) and matrix effects (maximum deviation 8.6%) were in the same range as for the respective analytes. Since the decrease in absolute peak areas was less than 20% of the initial value for all enantiomers, processed samples were stable for at least 63 h. Concentrations of amphetamine enantiomers were stable for the observed period of 10 months. The mean loss was 1.9 ng/mL for (R)-amphetamine and 1.0 ng/mL for (S)-amphetamine (median +0.1 and −0.5 ng/mL, respectively). Stability of amphetamine (R)/(S) concentration ratios is shown as a Bland–Altman plot in Figure 2. Mean deviation was 0.01, and deviations > ±1.96 SD were only found for one sample with rather low concentrations (total amphetamine concentration 45.2 ng/mL, mean ratio 2.01) and in two samples with concentrations at the upper end of the calibration range.

A slight freeze–thaw instability was found for both enantiomers of amphetamine (median loss each 7%), ranging from a (theoretical) 5% gain to a 16% loss. Maximum deviation of amphetamine (R)/(S) concentration ratios was 0.11 (median 0.01).

### 2.2. Forensic Serum Samples

**Figure 3:**
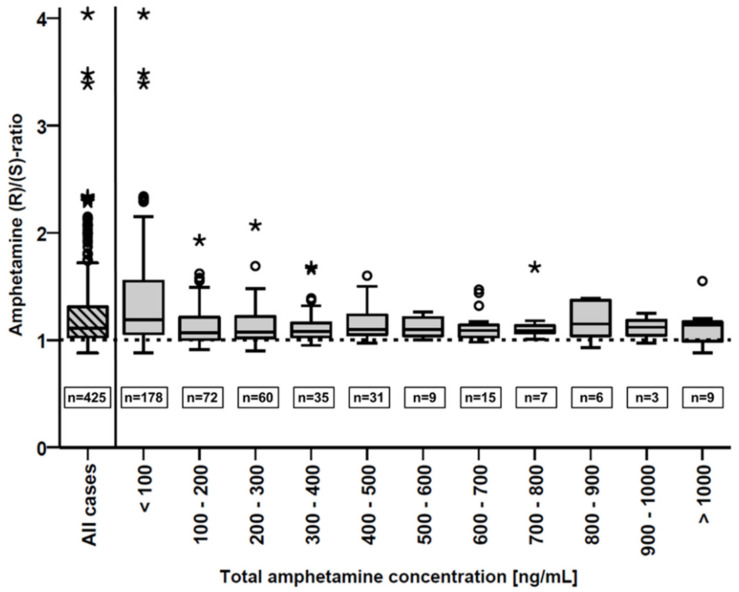
Boxplots of the determined amphetamine (R)/(S) ratios within subgroups of total amphetamine concentration. The striped left box represents the whole sample collective (n = 425). Horizontal lines represent the median, boxes represent the range between the lower and upper quartiles of the subgroups (interquartile range). Whiskers represent all samples within ±1.5 times the interquartile range. Outliers with (R)/(S) ratios greater than median ± 1.5 times the interquartile range are presented as circles (o). Extreme values with deviations greater than ±3 times the interquartile range are presented as asterisks (*).

Within the 425 cases examined, concentrations up to 784 ng/mL were found for (R)- and 887 ng/mL for (S)-amphetamine (same case, total concentration 1670 ng/mL). The calculated (R)/(S) concentration ratios were between 0.88 and 4.04 (mean 1.23, median 1.11). Ratios < 1.00 were observed in 75 cases (18%). The subject who exhibited an (R)/(S) ratio of 4.04 had serum concentrations of 8.6 and 2.1 ng/mL for (R)- and (S)-amphetamine, respectively. The boxplots in Figure 3 show the distribution of (R)/(S) concentration ratios within the entire collective and staggered by groups of total amphetamine concentrations (100 ng/mL intervals). While for total concentrations of <100 ng/mL the mean (R)/(S) ratio is significantly (one-way ANOVA, *p* < 0.001) higher than for the other groups (1.36 vs. 1.14), no clear trend can be observed for higher concentrations.

4-Hydroxyamphetamine was detected in 179 out of 425 cases (42%). In 100 cases, only the (R)-enantiomer could be detected; in another 71 cases, both enantiomers were found, while the (S)-enantiomer was detected alone in only 8 cases. The maximum concentrations found were 7.7 ng/mL for (R)- and 2.8 ng/mL for (S)-4-hydroxyamphetamine. Median concentrations were 1.2 and 0.8 ng/mL for the (R)- and the (S)-enantiomer, respectively. Median concentrations of (R)- and (S)-4-hydroxyamphetamine relative to their precursors (R)- and (S)-amphetamine were 0.8% and 0.5%, respectively. (R)/(S) concentration ratios of 4-hydroxyamphetamine ranged from 0.93 to 4.21 (median 2.19).

In 176 cases (41%), (1R,2S)-norephedrine was found alone (maximum concentration 7.2 ng/mL, median 1.0 ng/mL). Within the positive cases, the relative (1R,2S)-norephedrine concentration in relation to (S)-amphetamine was up to 5.4% (mean 0.9%, median 0.7%).

(1S,2R)-Norephedrine could only be detected in 11 cases (maximum concentration 1.6 ng/mL), 9 times together with (1R,2S)-norephedrine. Since (R)-amphetamine is not subjected to β-hydroxylation [31], (1S,2R)-norephedrine is not a metabolite of amphetamine. Therefore, detection of this enantiomer can only be explained by the use of other drugs (e.g., drugs containing racemic norephedrine or the interfering diastereomer cathine), for which reason the corresponding cases were excluded from further interpretation. While none of the herein detectable metabolites were detected in 42% of cases (n = 179), both 4-hydroxyamphetamine (any enantiomer) and (1R,2S)-norephedrine were detected in 26% of the cases (n = 109). In each 16% of cases, either 4-hydroxyamphetamine (n = 70) or (1R,2S)-norephedrine (n = 67) was detected next to amphetamine.

**Figure 4:**
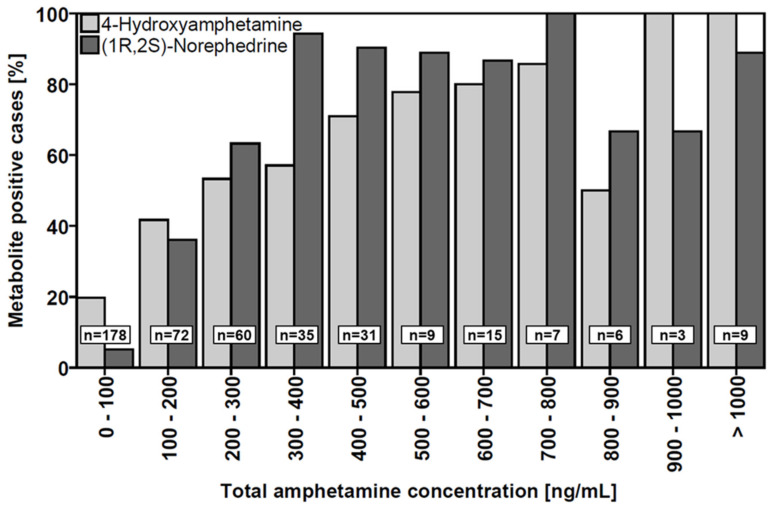
Histograms for relative frequency of 4-hydroxyamphetamine and (1R,2S)-norephedrine positive cases (at least 0.5 ng/mL of any enantiomer) within different total amphetamine concentration ranges. For both 4-hydroxyamphetamine and (1R,2S)-norephedrine, there was a positive correlation between positive cases and the total amphetamine concentration (one-tailed Spearman’s Rho and Kendall’s Tau each *p* = 0.000).

**Figure 5:**
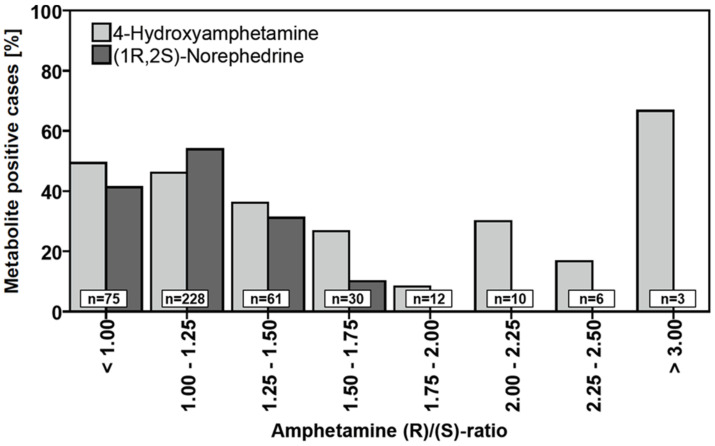
Histograms for relative frequency of 4-hydroxyamphetamine (any enantiomer) and (1R,2S)-norephedrine positive cases (each at least 0.5 ng/mL) within amphetamine^®^/(S) concentration ratio groups. For both 4-hydroxyamphetamine and (1R,2S)-norephedrine, there was a negative correlation between 4-hydroxyamphetamine positives and amphetamine (R)/(S) concentration ratios (one-tailed Spearman’s Rho and Kendall’s Tau each *p* < 0.001). There were no cases with (R)/(S) concentration ratios between 2.50 and 3.00.

Qualitative detection of 4-hydroxyamphetamine and (1R,2S)-norephedrine significantly correlated with staggered groups of total amphetamine concentration (100 ng/mL intervals, one-tailed Spearman’s Rho and Kendall’s Tau each *p* = 0.000, Figure 4). While (1R,2S)-norephedrine was very rarely detected at total amphetamine concentrations below 100 ng/mL (5.1%), it was found in the vast majority of samples with total amphetamine concentrations above 300 ng/mL. In contrast, at least one enantiomer of 4-hydroxyamphetamine was found in almost 20% of cases even at total amphetamine concentrations below 100 ng/mL. The increase in detection frequency with increasing amphetamine concentrations is not as rapid as in the case of (1R,2S)-norephedrine. In over 70% of cases with total amphetamine concentrations above 400 ng/mL, at least one enantiomer of norephedrine was detected. This was not the case for amphetamine concentrations between 800 and 900 ng/mL, where norephedrine was detected in only 50% of the cases, although the total number of cases in this group was very limited (n = 6). Moreover, qualitative detection of 4-hydroxyamphetamine and (1R,2S)-norephedrine significantly correlated (negatively) with staggered groups of (R)/(S) concentration ratios (0.25 intervals, one-tailed Spearman’s Rho and Kendall’s Tau each *p* < 0.001, Figure 5). While the metabolites could be detected in at least 40% of cases at (R)/(S) ratios below 1.25, detectability decreased at higher ratios. (1R,2S)-Norephedrine was not detectable at amphetamine (R)/(S) ratios above 1.75. In general, a similar trend was observed for the enantiomers of 4-hydroxyamphetamine, although even at ratios above 1.75 (n = 31) at least one of the two enantiomers was detectable in a total of 7 cases.

Quantitatively, (1R,2S)-norephedrine concentrations showed correlations (one-tailed Pearson) with both total amphetamine concentration (r = 0.507, *p* = 0.000) and the concentration of the parent compound (S)-amphetamine (r = 0.514, *p* = 0.000). In contrast, however, there was no quantitative correlation with the (R)/(S) concentration ratio (r = 0.053, *p* = 0.242).

### 2.3. Cases with Self-Reported Consumption Time

**Figure 6:**
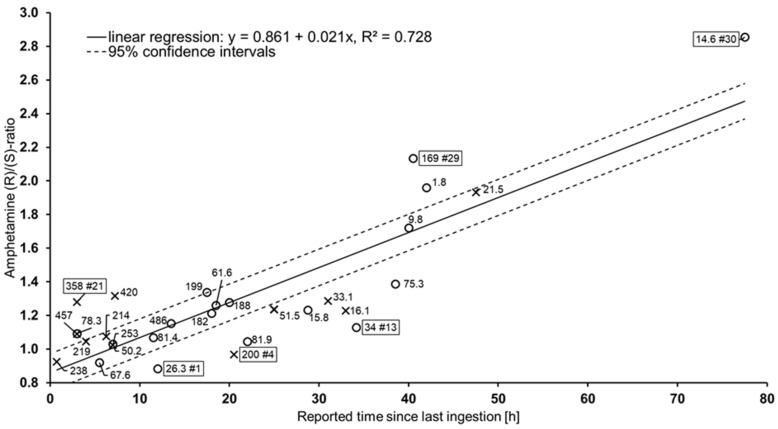
Plot of the determined amphetamine (R)/(S) ratios versus the reported time since last ingestion according to the patients’ statements (n = 30). Cases with reported binge consumption (further consumption of amphetamine within the last 24 h) are marked as crosses (x); all other cases are presented as circles (o). Labels show the total (racemic) amphetamine concentration of each patient in ng/mL. For some patients, the case number is also given with a number sign (#). Linear regression (solid line) was done using the method of least squares. Broken lines show 95% confidence intervals.

Within the psychiatric patient collective, concentrations up to 261 and 226 ng/mL (same case, total concentration 487 ng/mL) were found for (R)- and (S)-amphetamine, respectively. The reported times after last consumption were between 0.75 and 77.5 h. Calculated (R)/(S) concentration ratios were between 0.88 and 2.86 (median 1.22). Twelve patients (40%) reported at least one other use within 24 h prior to the last use, which was further considered as ‘binge’ consumption. These patients are discussed separately. The route of consumption was reported by 15 patients (50%). While one patient reported oral consumption, the majority (n = 12) consumed exclusively nasally. One patient each reported nasal/oral and nasal/intravenous use.

(1R,2S)-Norephedrine was detected in 12 cases, reaching concentrations up to 7.0 ng/mL (reported time 4.0 h, (R)/(S) ratio 1.09), while in the other cases concentrations were below 1.8 ng/mL. (1S,2R)-Norephedrine was absent in all samples. The maximum reported time with detection of (1R,2S)-norephedrine was 38.5 h ((R)/(S) ratio 1.39). None of the 4-hydroxyamphetamine enantiomers could be detected in a single case. Table 2 shows the determined concentrations and (R)/(S) ratios for all (n = 30) cases with self-reported consumption time. For further interpretation, the (R)/(S) concentration ratios were plotted against the reported last consumption time (Figure 6). Linear regression (method of least squares) was carried out and showed high correlation (R^2^ = 0.728) between time intervals and (R)/(S) ratios. Furthermore, 95% confidence intervals were calculated. The slope of the linear fit was 0.021 h^−1^, and the y-intercept was at an (R)/(S) ratio of 0.861. There were both patients with rather high serum concentrations and longer time to last consumption (e.g., case 29) and patients who reported having consumed a few hours earlier but showed rather low concentrations (e.g., case 12).

## 3. Discussion

### 3.1. Method Validation

In the present study, a total of 425 forensic serum samples and 30 serum samples from psychiatric patients, stating their last time of consumption, were enantioselectively analyzed for amphetamine and specific metabolites. A chiral LC-MS/MS method for detection of amphetamine, norephedrine and hydroxyamphetamine was developed, validated and applied for analysis of the specimens. To the best of our knowledge, this study is the first to enantioselectively detect amphetamine and metabolites in a comprehensive collective of serum samples and evaluate them from a forensic perspective.

The method was sensitive and selective, and all analytes met all acceptance criteria of the validation. At the high concentration level, recovery was slightly higher than at the low level, although it was rather low for all analytes, especially for norephedrine and hydroxyamphetamine. Amphetamines are known to be volatile compounds that may be lost during evaporation, especially at high temperatures [37,38]. Therefore, the extracts were evaporated (nearly) to dryness in as short a time as possible at only 40 °C so that reproducible results were ensured. Since all validation parameters were met despite the comparatively low recovery, the sample preparation method used was considered suitable.

For this study, the detected interference of (1S,2R)-norephedrine with cathine is not relevant, since this enantiomer is not a metabolite of amphetamine. The results for long-term and freeze–thaw stability for amphetamine were in line with other (chiral) studies [39,40,41]. The experiments of Beyer et al. do not confirm the slight freeze–thaw instability found here [42]. Deviations in (R)/(S) concentration ratios after reanalysis were within the measurement inaccuracies. Complete stability of the ratios is therefore assumed, which enables forensic interpretation even after longer storage time.

### 3.2. Forensic Serum Samples

(R)/(S) concentration ratios of phenethylamines consumed as a racemic mixture have been discussed to give a hint for the last time of consumption due to an enantioselective metabolism [34,35,36]. For amphetamine, we showed that in Rhineland-Palatinate it is used exclusively as the racemate [43], and it can be assumed that this is true worldwide due to non-stereoselective pathways of synthesis [35,44,45].

The amphetamine (R)/(S) ratios calculated in the sample collective are consistent with the use of racemic amphetamine, taking into account the shorter elimination half-life of (S)-amphetamine [28,29]. The median (R)/(S) ratio in this study was 1.11, which was significantly lower than the median of 1.69 found by Hess et al. in a similar study (n = 28) [35]. The maximum (R)/(S) ratio of 4.50 found there is similar to that of the present study (4.04). In the study of Hess et al., (R)/(S) ratios below 1.00 were only detected in combination with methamphetamine intake (which is mostly consumed as pure (S)-methamphetamine, and thus only (S)-amphetamine is formed as a metabolite), while in our study, ratios between 0.88 and 1.00 were found in a total of 75 samples (18%). Peters et al. found (R)/(S) ratios ranging from 0.97 to 1.66 (mean 1.15, median 1.10) in a total of 23 quantifiable samples with exclusive amphetamine use [39]. Moreover, in a controlled oral intake study by Wan et al. (10 mg racemic amphetamine, n = 4), (R)/(S) ratios ranging from 0.84 to 1.18 were observed during the absorption phase [29]. The occurrence of (R)/(S) ratios below 1.00 is also supported by the studies of Caras and Sharpe, which found higher mean peak serum concentrations of (S)-amphetamine while the times of peak serum concentrations of both enantiomers were similar [28]. Furthermore, in a study with controlled oral intake of racemic 4-fluoroamphetamine (4-FA), (R)/(S) ratios below 1.00 were observed during the absorption phase (partially even beyond) [36]. Havnen et al. also found (R)/(S) ratios around 1.00, although all samples were from patients under treatment with prescribed nonracemic amphetamine (n = 61), and the results are therefore hardly comparable [46]. Even in the present study, an additional intake of medicinal drugs, as also described by Musshoff et al. [47], cannot be basically ruled out. In Germany, (S)-amphetamine (Attentin) and its precursor lisdexamfetamine (Elvanse) can be prescribed for treatment of attention deficit hyperactivity disorder (ADHD). More seldom, formulations of racemic amphetamine sulfate can be prescribed [35,47]. Adderall, which is approved in the United States, contains approximately a 3:1 mixture of (S)- and (R)-amphetamine [48]. In the case of exclusive use of illicit (racemic) amphetamine, serum (R)/(S) ratios below 1.00 could be explained, e.g., by a faster uptake of (S)-amphetamine or a more pronounced first-pass effect for (R)-amphetamine. In general, especially at lower concentrations, measurement inaccuracies must also be taken into account.

The significantly higher mean amphetamine (R)/(S) ratio at total concentrations below 100 ng/mL indicates an advanced elimination of (S)-amphetamine, which is consequently associated with lower absolute amphetamine concentrations. However, at concentrations above 100 ng/mL, this trend continued only partially. Outliers with higher (R)/(S) ratios also occurred at higher total amphetamine concentrations. This can probably be explained mainly by repeated intake (binge use), which is common among chronic amphetamine users [49]. However, high (R)/(S) ratios are generally less frequently found at higher amphetamine concentrations. Moreover, even at total amphetamine concentrations below 100 ng/mL, there are many samples exhibiting rather low (R)/(S) ratios. Since dosages can differ considerably between occasional (recreational) users and chronically dependent users due to habituation effects [16], recent consumption should generally be considered even at low concentrations when (R)/(S) ratios are rather small.

Amphetamine is metabolized to hydroxyamphetamine by liver monooxygenases and about 3–7% is excreted as such [30,31,32,33]. The fact that in almost all cases either (R)-4-hydroxyamphetamine alone or both enantiomers were found is consistent with the observation by Dring et al. in humans, that (R)-amphetamine is metabolized to 4-hydroxyamphetamine to a greater extent than (S)-amphetamine or the racemate is [33]. Two to three percent of amphetamine is excreted renally as norephedrine, and the metabolism is probably catalyzed by dopamine β-hydroxylase, which is located in the central and peripheral nervous system [30,31,32]. The relatively low serum concentrations of (1R,2S)-norephedrine found in the present study (median 1.0 ng/mL) were expected for this minor metabolite. The detection of (1S,2R)-norephedrine in only 11 cases is consistent with the observation that β-hydroxylation occurs selectively only for (S)-enantiomers [30,31]. Since the results in the corresponding cases are distorted by the use of medication or other drugs, the corresponding cases were not considered for further evaluation.

(1R,2S)-Norephedrine and 4-hydroxyamphetamine were detected in an almost equal number of cases (n = 176 vs. 179), with a relatively large intersection of samples in which both metabolites were found (n = 109). Both 4-hydroxyamphetamine and (1R,2S)-norephedrine showed a statistically significant trend of increasing detectability with higher amphetamine concentrations. The increase in detectability with increasing amphetamine concentration was significantly steeper for (1R,2S)-norephedrine. In contrast to (1R,2S)-norephedrine, enantiomers of 4-hydroxyamphetamine were detected considerably more frequently at amphetamine concentrations below 100 ng/mL. Overall, 4-hydroxyamphetamine-positive cases are thus more widely distributed with regard to absolute amphetamine concentrations. Regarding the determined amphetamine (R)/(S) ratios, a statistically significant trend of more frequent detection at lower ratios was observed for both 4-hydroxyamphetamine and (1R,2S)-norephedrine. Assuming that the shorter elimination half-life of (S)-amphetamine leads to increasing (R)/(S) ratios over time and that low ratios therefore indicate a more recent ingestion, (1R,2S)-norephedrine seems to be more suitable as a qualifying criterion since, unlike hydroxyamphetamine, it was not detectable at (R)/(S) ratios over 1.75. In addition, (1R,2S)-norephedrine was less frequently detectable at lower amphetamine concentrations. Taken together, these results indicate that (1R,2S)-norephedrine offers higher potential than 4-hydroxyamphetamine to foster forensic interpretations, although in this case the ingestion of other substances must be taken into account when performing achiral analysis. Thus, according to the results of this study and under assurance of appropriate analytical sensitivity, the detection of (1R,2S)-norephedrine could strengthen a suspicion of recent amphetamine consumption.

### 3.3. Cases with Self-Reported Consumption Time

The observed time period, which for most patients was no longer than 48 h, is equivalent to the typical detection time for amphetamine in serum samples [50]. The maximum serum concentration determined was significantly lower than in the forensic sample collective, while the median was slightly higher. The maximum determined (R)/(S) ratio was slightly lower for the psychiatric patients. Since this collective comprises dependent users who voluntarily undergo psychiatric treatment, the incidence of binge use was very high at 40%. Both the maximum concentration of (1R,2S)-norephedrine and the detection frequency were approximately identical with those of the forensic serum samples. On the other hand, it remains unclear why 4-hydroxyamphetamine was not detected in any case, which is in contrast to the forensic samples.

Amphetamine intoxications are one of the most common drug-induced impairments, which is particularly relevant in driving under the influence of drugs (DUID) [3,47,51]. Especially in this context, information about the time of use and the current influence of stimulants is critical [3,51]. On the basis of the total amphetamine concentration, as it is typically determined, such conclusions cannot be made [15].

The determination of (R)/(S) concentration ratios and the evaluation of the respective time course have already been proposed in some studies. In studies of stereoselective metabolism of 3,4-methylenedioxy-N-methylamphetamine (MDMA) by Fallon et al. and Steuer et al. (oral intake of 40 and 125 mg racemate, respectively), mean (R)/(S) concentration ratios increased to approximately 8 within 24 h (slope 0.33 h^−1^) [34,52].

The increase in MDMA (R)/(S) ratios is thus significantly more rapid than that found for amphetamine in the present study. In our previous study on 4-fluoroamphetamine (4-FA) metabolism, slopes for individual subjects ranged from 0.023 to 0.157 h^−1^ [36]. This faster increase in the MDMA and 4-FA (R)/(S) ratios with time is due to the shorter elimination half-lives and the larger relative difference (approx. factor 2).

The human serum half-lives of the amphetamine enantiomers were determined in two studies. Wan et al. determined (with oral intake of 10 mg racemate) serum half-lives of 23.7 and 7 h under alkaline and 7.7 and 6.8 h under acidic urine conditions for (R)- and (S)-amphetamine, respectively [29]. Assuming first-order elimination kinetics [53] and a mean (R)/(S) concentration ratio of 1.00 at the start of elimination [29], the theoretical slopes of the (R)/(S) ratios with time can be calculated from the serum half-lives using a linear fit. For alkaline urine conditions, slopes of 0.013 and 0.018 h^−1^ are calculated for 24 and 72 h, respectively. For acidic urine conditions, the theoretical slopes are 0.014 and 0.019 h^−1^.

Using the serum half-lives determined by Caras et al. (oral intake of 30 mg racemate) of 13.4 and 10.8 h for (R)- and (S)-amphetamine, respectively [28], slopes of 0.015 and 0.020 h^−1^ are obtained over 24 and 72 h, respectively. In this context, it should be noted that the theoretical slope of the (R)/(S) ratios increases with time, and the linear fit is thus an approximation. However, in the calculated examples, the absolute error of the linear fit for the (R)/(S) ratios after 72 h is only 0.11 at maximum. The slopes calculated theoretically are consistently slightly lower than the slope of 0.021 h^−1^ determined in this study.

Within the observed collective, some outliers far outside the 95% confidence intervals appeared. Subject 1 was found to have the lowest (R)/(S) ratio of the entire collective (0.88), although the last consumption was self-reported to be as long as 12 h earlier. Subject 4, who reported the last consumption 20.5 h earlier, exhibited a very low ratio of 0.97. Subject 13 reported last use 34 h earlier and exhibited a ratio of only 1.13, although the absolute amphetamine concentration of 34 ng/mL was rather low. There were also outliers located above the 95% confidence interval. In general, higher (R)/(S) ratios can be explained by a binge-associated enrichment of the (R)-enantiomer. This could explain, for example, the comparatively higher (R)/(S) ratio (1.28) of Subject 21. Subject 29 showed a high (R)/(S) ratio (2.14), although a rather high total amphetamine concentration of 169 ng/mL was present in serum. Thus, the indicated longer past use (40.5 h) seems plausible despite the considerable amphetamine concentration. This indicates that the respective dose consumed must have been above the average amount.

In the studies by Fallon and Losacker, high interindividual variances of the (R)/(S) ratios were found for MDMA and 4-FA, respectively, which gradually increased over time. Particularly in the 4-FA study, the (R)/(S) ratio for one subject was still below 1.00 even after 12 h. Considering this, the scatter of outliers found in the present study appears moderate. After oral ingestion of (S)-amphetamine, peak effects are reached after about 3 h [54], and the acute phase lasts for about 6 h [11].

The upper limit of the 95% confidence interval after 6 h is at an (R)/(S) ratio of 1.09. Ratios above this cut-off would therefore suggest the absence of acute effects.

Subject 21, however, exhibited an (R)/(S) ratio above this cut-off, although the last consumption was indicated 3 h earlier. One explanation for this could be the reported binge consumption. In contrast, (R)/(S) ratios below the cut-off of 1.09 were observed up to 22.0 h after the claimed last consumption.

Despite the variation of the determined ratios with the reported time, chiral amphetamine analysis can allow important conclusions in certain cases. Thus, for Subject 29, it can be assumed that acute effects had passed, although this would not necessarily have been indicated by looking at the total amphetamine concentration. It must of course always be taken into account that exhaustion reactions such as fatigue, which occur in the subacute phase, can also restrict the driving ability [3,15].

For the forensic toxicological expert, the evaluation of (R)/(S) ratios must take into account whether, for example, a more recent consumption can incriminate or exonerate the defendant. For this purpose, it should be asked when exactly the last consumption had taken place. Furthermore, it is of great relevance whether it was a single or repeated use. In the case of binge use, the enrichment of (R)-amphetamine in the blood leads to higher (R)/(S) ratios, which would result in an overestimation of the time difference to consumption. However, extreme shifts in the (R)/(S) ratio should generally not be expected with binge use. With a longer time interval between two doses, the serum concentrations remaining from the first consumption are expected to be minor compared to the total concentration, and with a short time interval, the ratio should be close to 1.00 anyway.

The observed collective consisted of dependent psychiatric inpatients, some of which were also abusing other substance groups such as opiates, benzodiazepines or cannabis. Higher dosages due to habituation effects, liver damage and metabolic effects due to enzyme induction or inhibition therefore cannot be ruled out. The enzyme CYP2D6, which is involved in the metabolism of amphetamine and is known for its stereoselectivity, exhibits a genetic polymorphism [17,18,55,56]. Consequently, high interindividual differences have to be taken into account. Poor metabolizers lacking CYP2D6 could thus exhibit low (R)/(S) ratios over a longer period of time. Furthermore, consumption of very high doses or concomitant use of other drugs may lead to saturation of CYP2D6 so that metabolism is taken over by other (less stereoselective) enzymes. Illegal amphetamine is present exclusively in racemic form [4,43,57]. For forensic interpretation, therefore, the (additional) use of (S)-amphetamine-containing drugs must be excluded, as this medication would lead to an underestimation of the time difference to consumption.

## 4. Materials and Methods

### 4.1. Material

(RS)-Amphetamine and racemic norephedrine-d3 were purchased from LGC (Wesel, Germany). (RS)-Amphetamine d11, (S)-amphetamine, (R)-amphetamine, racemic norephedrine and (RS)-4-hydroxyamphetamine were obtained from Lipomed (Weil am Rhein, Germany). Methanol (HPLC-grade) was purchased from Thermo Fisher Scientific (Dreieich, Germany). Water (LC-MS-grade), acetonitrile, dichloromethane, acetone, isopropanol, glacial acetic acid, potassium dihydrogenphosphate and potassium hydroxide were obtained from Carl Roth (Karlsruhe, Germany). LC-MS-grade ammonia solution (25%) and ammonium bicarbonate were purchased from Merck (Darmstadt, Germany). All chemicals were at least of analytical grade. Mixed-mode cation-exchange solid-phase extraction columns (HF BE-CERTIFY, 300 mg, 3 mL) were purchased from Agilent (Waldbronn, Germany). Round-robin tests ‘BTMF—Drugs in serum’ containing certified racemic amphetamine were acquired from Arvecon (Walldorf, Germany). Blank (drug-free) serum was provided by the blood bank of the University Medical Center of the Johannes Gutenberg University Mainz.

### 4.2. Sample Preparation

Two hundred microliters of serum was spiked with 10 µL internal standard solution (containing 2 µg/mL racemic amphetamine-d11 and 2 µg/mL racemic norephedrine-d3 in methanol). Samples with concentrations outside of the calibration range were diluted with water to reach appropriate concentrations and reanalyzed. Six hundred microliters of acetonitrile was added for protein precipitation. After mixing, samples were centrifuged for 10 min at 1200× *g*. Subsequently, 6 mL of phosphate buffer solution (0.1 M, pH 6) was added to the supernatants. For solid-phase extraction (SPE), cartridges were equilibrated with 2 × 3 mL methanol followed by 2 × 2 mL water. Samples were applied and washed with 2 × 2 mL water and 2 × 2 mL water/methanol (80/20, *v*/*v*). After addition of 1 mL acetic acid (0.1 M), cartridges were centrifuged for 10 min at 1000× *g*. After flushing with 3 mL dichloromethane/acetone (50/50, *v*/*v*), elution was done using 3 mL dichloromethane/isopropanol/ammonia solution (80/20/4, *v*/*v*/*v*). The extracts were evaporated to dryness under a gentle stream of nitrogen at 40 °C. For analysis, residues were redissolved in 50 µL of methanol.

### 4.3. Chiral LC-MS/MS Instrumentation and Analytical Parameters

Enantioselective quantification was done using a liquid chromatography–tandem mass spectrometry (LC-MS/MS) system from Agilent (Waldbronn, Germany). Chromatography was conducted using a 1290 Infinity II LC system, coupled via Jet Stream interface (ESI) to a 6495C triple-quadrupole mass spectrometer. Chiral chromatographic separation was achieved using a polysaccharide-based chiral Lux 3 µm AMP 150 × 3.0 mm analytical column, guarded with a Lux AMP 4 × 2.0 mm security guard cartridge (both Phenomenex; Aschaffenburg, Germany). Injection volume was 1 µL. The mobile phase consisted of 5 mM ammonium bicarbonate solution adjusted to pH 11 by addition of ammonia solution (A) and methanol (B). Holding a temperature of 30 °C and a flow rate of 0.35 mL/min, chromatography was performed isocratically at 60% B for 13 min. For column washing, B was increased to 95% within 0.5 min, held for 3 min and decreased to 60% within 0.5 min followed by re-equilibration during a post-time of 2 min (total run time 19 min). Electrospray parameters were as follows: gas flow 11 L/min at 200 °C; nebulizer 15 psi, sheath gas flow 12 L/min at 400 °C; capillary voltage +3500 V. Analytes were detected by multiple reaction monitoring (MRM) mode using the following transitions (*m*/*z*, collision energy in parentheses, target ion underlined): amphetamine 136.1 → 91.0 (17 eV), 119.0 (5 eV), 65.0 (45 eV); amphetamine-d11 147.2 → 98.0 (21 eV), 130.1 (5 eV), 70.1 (45 eV); norephedrine 152.1 → 134.0 (9 eV), 117.0 (17 eV), 115.0 (25 eV); norephedrine-d3 155.1 → 137.1 (9 eV), 119.1.0 (21 eV), 117.1 (29 eV); 4-hydroxyamphetamine 152.1 → 107.0 (21 eV), 135.0 (5 eV), 77.0 (45 eV). For quantification of each enantiomer, corresponding enantiomers of the internal standards were used. Norephedrine-d3 was used as internal standard for quantification of 4-hydroxyamphetamine. Data evaluation was done using Agilent Mass Hunter Workstation Software (Version B.09.00). Identification of the elution order of amphetamine enantiomers was conducted by injection of enantiopure standard solutions. For all other experiments, racemic solutions were used as analytical standards. Due to the lack of enantiopure standards for norephedrine and 4-hydroxyamphetamine, elution order of these metabolites could not be determined but was deduced. Since no racemization of these substances takes place in the human body [58], the absolute configuration (at the 2-position) is retained. Accordingly, when enantiopure formulations such as dexamphetamine or lisdexamfetamine (Elvanse) are ingested, only (1R,2S)-norephedrine and (S)-4-hydroxyamphetamine are formed as metabolites. By analyzing respective serum samples, it could be shown that the mentioned metabolites of (S)-amphetamine elute second, as does their parent substance.

### 4.4. Method Validation

The method was validated according to an international forensic guideline [59]. Validation parameters were selectivity, linearity of calibration, analytical limits, accuracy (bias), interday precision, recovery, matrix effects and processed sample stability. Statistical evaluation was performed using Valistat 2.00.1 (Arvecon; Walldorf, Germany).

Evaluation of selectivity was done by analyzing drug-free serum samples of 10 different donors (blank samples) and two drug-free samples after addition of internal standard (zero samples). A 100 ng/mL solution of cathine ((1S,2S)-norpseudoephedrine), which is a diastereomer of norephedrine, was injected to check for interferences. Linearity of calibration was tested with three calibration series, which included the following calibration points: 0.5, 2.5, 5, 10, 25, 50, 75, 100, 150 and 250 ng/mL. LOD was determined by means of signal-to-noise ratio (S/N, >3). LLOQ was established for the lowest calibrator by means of a 5-fold analysis requiring less than 20% RSD for precision and less than ± 20% for bias. Accuracy (bias), interday precision, recovery and matrix effects were determined at low (20 ng/mL) and high (125 ng/mL) concentrations relative to the calibration range. Quality control working solutions and calibration working solutions were prepared separately from different stock solutions. For determination of accuracy and interday precision, samples of each concentration were prepared and analyzed on six different days. Accuracy of calculated concentrations was accepted within a maximum bias of 15%. Interday precision was calculated as RSD (%) from 6-fold determinations. Furthermore, external serum round-robin tests containing certified racemic amphetamine were examined for quality control. Recovery and matrix effects were determined according to the recommendations of Matuszewski et al. [60]. Processed sample stability was evaluated at low and high concentrations by pooling and subsequently splitting six samples of each concentration, followed by repeated analysis over 63 h. For determination of the long-term stability of amphetamine enantiomer concentrations and stability of amphetamine (R)/(S) concentration ratios, 50 amphetamine-positive forensic samples were reanalyzed 10 months after the first analysis. For amphetamine, freeze–thaw stability was determined by reanalysis of spiked samples at low and high concentration levels (20 and 250 ng/mL, respectively) after three freeze–thaw cycles. Additionally, three real forensic samples were reanalyzed after three cycles.

### 4.5. Investigated Collectives

#### 4.5.1. Forensic Serum Samples

A total of 425 amphetamine-positive forensic serum samples were analyzed. All samples originate from the year 2020 and were sent to the Department of Forensic Toxicology at the Institute of Forensic Medicine in Mainz, Germany, from police stations in the federal state of Rhineland-Palatinate for toxicological analysis. After centrifugation, the serum samples were stored at −20 °C. Samples from individuals reporting consumption of pharmaceutical drugs containing (S)-amphetamine or (S)-amphetamine precursors (e.g., Attentin or Elvanse) or other enantiomeric compositions of amphetamine (e.g., Adderall) were excluded to prevent the data from being distorted. Cases positive for methamphetamine were also excluded, since amphetamine represents the main metabolite and this would therefore bias the results [4].

#### 4.5.2. Cases with Self-Reported Consumption Time

A total of 30 serum samples with self-reported consumption of amphetamine within the last 48 h were analyzed. The samples were collected from inpatients at the Department of Psychiatry and Psychotherapy, University Medical Center Mainz, and at the Clinic and Polyclinic for Psychiatry and Psychotherapy, University Hospital Bonn. Ward 6 (not protected) of the University Hospital Mainz is specialized in the treatment of patients with various types of addiction. The “Freud” ward (protected) of the University Hospital Bonn focuses on addiction medicine for qualified detoxification treatments, especially for opiate-dependent patients. However, in both clinics, admission and treatment are voluntary. Upon admission to both wards, patients are asked about their substance abuse (in particular, substances abused, last use, route of consumption and frequency of use). The collected blood samples were stored in serum tubes at 4 °C and kept at −20 °C after centrifugation.

## 5. Conclusions

In the present study, serum samples from two different collectives were stereoselectively analyzed for the enantiomers of amphetamine and metabolites. Increasing amphetamine (R)/(S) ratios were observed over time. The metabolites were found significantly more frequently at lower amphetamine (R)/(S) ratios indicating recent consumption. The advantages of a stereoselective analysis were discussed regarding forensic questions. For the future, an expansion of the patient collective should be targeted in order to achieve a more robust data basis. Generally, a controlled ingestion study would be superior, although this is hardly feasible due to ethical factors.

## Figures and Tables

**Table 1:** Validation results for the enantiomers of amphetamine (AM), norephedrine (NE) and 4-hydroxyamphetamine (4OH-AM) in serum.

Concentration	Analyte	Accuracy (Bias) n = 6	Interday Precision n = 6	Recovery n = 6	Matrix Effects n = 6
(ng/mL)		(%)	(%)	(%)	(%)
20	(R)-AM	−1.7	0.4	28.1 ± 3.2	86.5 ± 6.5
20	(S)-AM	4.5	4.3	28.4 ± 3.9	87.3 ± 6.7
125	(R)-AM	3.3	2.7	30.3 ± 3.3	106 ± 7.3
125	(S)-AM	7.3	2.5	32.3 ± 3.6	105 ± 7.7
20	(1S,2R)-NE	3.4	4.6	10.3 ± 1.2	99.4 ± 8.4
20	(1R,2S)-NE	−1.2	1.1	10.7 ± 1.3	97.8 ± 8.9
125	(1S,2R)-NE	5.2	6.0	13.4 ± 1.6	112.2 ± 9.3
125	(1R,2S)-NE	−0.5	8.4	13.5 ± 1.7	112.5 ± 8.4
20	(R)-4OH-AM	1.8	12.8	10.0 ± 0.9	78.7 ± 10.3
20	(S)-4OH-AM	−0.3	17.6	10.4 ± 1.2	98.4 ± 7.7
125	(R)-4OH-AM	0.2	9.7	14.5 ± 3.4	85.5 ± 4.9
125	(S)-4OH-AM	1.4	13.7	14.6 ± 1.8	114.8 ± 6.7

**Table 2:** Summary of cases with self-reported consumption time.

Case	Binge	Δ*t*	(R)-AM	(S)-AM	Σ AM	(R)/(S)	(1R,2S)-NE
(ng/mL)		(h)	(ng/mL)	(ng/mL)	(ng/mL)		(ng/mL)
1		12.0	12.3	13.9	26.3	0.88	
2		5.5	32.4	35.2	67.6	0.92	
3	*	0.8	114	124	238	0.93	0.6
4	*	20.5	98.3	102	200	0.97	
5	*	7.0	128	125	253	1.02	1.2
6		7.0	25.5	24.7	50.2	1.03	
7		22.0	41.9	40.1	81.9	1.04	
8	*	4.0	112	107	219	1.05	0.6
9		11.5	42.1	39.3	81.4	1.07	
10	*	6.3	111	103	214	1.08	1.4
11	*	3.0	239	219	457	1.09	7.0
12		3.0	40.9	37.4	78.3	1.09	1.4
13		34.2	18.0	16.0	24.0	1.13	
14		13.5	261	226	487	1.15	1.8
15		18.0	100	82.4	182	1.21	1.4
16	*	33.0	8.9	7.2	16.1	1.23	
17		28.8	8.7	7.1	15.8	1.23	
18	*	25.0	28.5	23.1	51.5	1.23	0.9
19		18.5	34.3	27.2	61.6	1.23	1.0
20		20.0	105	82.4	188	1.28	
21	*	3.0	201	157	358	1.28	
22	*	31.0	18.6	14.5	33.1	1.29	
23	*	7.3	239	181	420	1.32	
24		17.5	114	85.1	199	1.34	0.7
25		38.5	43.8	31.5	75.3	1.39	0.9
26		40.0	6.2	3.6	9.8	1.72	
27	*	47.5	14.2	7.3	21.5	1.93	
28		42.0	1.2	0.6	1.8	1.96	
29		40.5	115	53.8	169	2.14	
30		77.5	10.3	3.6	13.9	2.86	

The table shows the patient-reported time interval between last consumption and blood collection (Δ*t*), as well as the determined concentrations of (R)-amphetamine ((R)-AM), (S)-amphetamine ((S)-AM), total amphetamine (Σ AM) and (1R,2S)-norephedrine ((1R,2S)-NE). Case numbers are assigned by ascending amphetamine (R)/(S) ratio. Asterisks (*) indicate patient-reported binge consumption (further consumption of amphetamine within the last 24 h prior to the reported last use).

## Data Availability

The data presented in this study are available in article.
